# 342. Multicenter Evaluation of Fosfomycin MIC Results for *Enterobacterales* Using EUCAST V11 Breakpoints (i.v. and oral for uncomplicated UTI only, E. coli) on MicroScan Dried Gram Negative MIC Panels

**DOI:** 10.1093/ofid/ofac492.420

**Published:** 2022-12-15

**Authors:** Omai Garner, Amanda Harrington, Sharon DesJarlais, Maria Traczewski, Denise Beasley, Jose Diaz, Regina Brookman, Zabrina Lockett, Jennifer Chau

**Affiliations:** University of California Los Angeles, Los Angeles, California; Loyola University Chicago, Maywood, Illinois; Loyola University & Medical Center, Maywood, Illinois; Clinical Microbiology Institute, Wilsonville, Oregon; Clinical Microbiology Institute, Wilsonville, Oregon; Beckman Coulter, Inc., West Sacramento, CA, California; Beckman Coulter, Inc., West Sacramento, CA, California; Beckman Coulter, Inc., West Sacramento, CA, California; Beckman Coulter, Inc., West Sacramento, CA, California

## Abstract

**Background:**

EUCAST V11 fosfomycin breakpoints for *Enterobacterales* (i.v. and oral for uncomplicated UTI only, E. coli) were evaluated against data from a multicenter clinical study on a MicroScan Dried Gram Negative MIC (MSDGN) Panel. MIC results were compared to results obtained with agar dilution reference prepared according to CLSI methodology.

**Methods:**

A total of 344 Gram negative clinical isolates including 191 *Enterobacterales* were tested using the turbidity and Prompt® methods of inoculation during the efficacy phase at three clinical sites. An evaluation was conducted by comparing MIC values obtained using the MSDGN panels to MICs utilizing the CLSI agar dilution reference. MSDGN panels were incubated at 35 ± 1^°^C and read on the WalkAway System, the autoSCAN-4 instrument, and read visually at 16-20 hours. Agar dilution plates were prepared according to CLSI methodology, incubated for 16-20 hours and read visually. EUCAST v11.0 fosfomycin breakpoints (mg/L) used for interpretation of MIC results were: i.v. for *Enterobacterales* ≤ 32 S, > 32 R and oral for uncomplicated UTI only, *E. coli* ≤ 8 S, > 8 R.

**Results:**

Essential agreement, categorical agreement and categorical errors were calculated compared to MIC results from agar dilution plates. Results for all efficacy isolates with turbidity inoculation and manual read are found in Table 1.

**Table 1:**
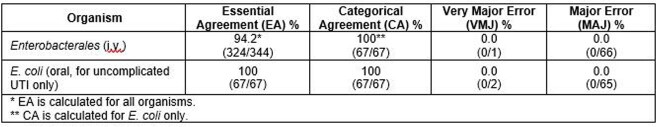
- Results

**Conclusion:**

This multicenter study showed that fosfomycin MIC results for i.v. for *Enterobacterales* and *E. coli* (oral, for uncomplicated UTI only) obtained with the MSDGN panel correlate well with MICs obtained using CLSI agar dilution reference using EUCAST interpretive criteria.

© 2022 Beckman Coulter. All rights reserved. All other trademarks are the property of their respective owners.

Beckman Coulter, the stylized logo, and the Beckman Coulter product and service marks mentioned herein are trademarks or registered trademarks of Beckman Coulter, Inc. in the United States and other countries.

**Disclosures:**

**Omai Garner, PhD**, Beckman Coulter, Inc.: Clinical trial data collection funded by Beckman Coulter, Inc. **Amanda Harrington, PhD**, Beckman Coulter, Inc.: Clinical trial data collection funded by Beckman Coulter, Inc.|bioMeriuex/BioFire: Grant/Research Support **Sharon DesJarlais, BS**, Beckman Coulter, Inc.: Clinical trial data collection funded by Beckman Coulter, Inc. **Maria Traczewski, BS**, Beckman Coulter, Inc.: Clinical trial data collection funded by Beckman Coulter, Inc. **Denise Beasley, BS**, Beckman Coulter, Inc.: Clinical trial data collection funded by Beckman Coulter, Inc. **Jose Diaz, BS**, Beckman Coulter, Inc.: Employee of Beckman Coulter **Regina Brookman, BS**, Beckman Coulter, Inc.: Employee of Beckman Coulter **Zabrina Lockett, PhD**, Beckman Coulter, Inc.: Employee of Beckman Coulter **Jennifer Chau, PhD**, Beckman Coulter, Inc.: Employee of Beckman Coulter.

